# Chemical Composition of Atmospheric Air in Nemoral Scots Pine Forests and Submountainous Beech Forests: The Potential Region for the Introduction of Forest Therapy

**DOI:** 10.3390/ijerph192315838

**Published:** 2022-11-28

**Authors:** Tomasz Dudek, Mariusz Marć, Bożena Zabiegała

**Affiliations:** 1 Department of Agroecology and Forest Utilization, University of Rzeszów, 35-601 Rzeszów, Poland; 2 Department of Analytical Chemistry, Faculty of Chemistry, Gdańsk University of Technology, 80-233 Gdańsk, Poland

**Keywords:** forest bathing, public health, terpenes, forest air, environmental quality

## Abstract

Studies show that forests are one of the main recreational destinations. This can be explained by their beneficial effects on the health of their visitors, which can be attributed to compounds from the terpene group. The aim of this research was to determine the chemical composition of air in the interiors of Nemoral Scots pine forests and submountainous beech forests, with the determination of compounds of the terpene group. Samples of organic compounds present in the air were collected with the use of Tenax TA sorbent tubes. The process of separation, identification, and determination of the extracted organic compounds was carried out with the use of the gas chromatography technique integrated with a flame ionization detector. Additional identification of the extracted compounds was carried out with the use of GC coupled with mass spectrometry. The most abundant group of compounds was the aliphatic hydrocarbons, both saturated (linear and branched) and unsaturated (terpenes). Carbonyl compounds were also found in the collected samples, but they constituted no more than 10% of all compounds present on the chromatograms. The concentrations of terpenes and terpenoids in the forest atmosphere varied from 10 to 74 µg·m^−3^, representing on average 33% of the total volatile organic compounds.

## 1. Introduction

Research conducted in different parts of the world shows that forests are one of the main recreational destinations for the population of urban agglomerations [1,2]. This is particularly true for the forests closest to the place of residence [3], which for the majority of the population are urban and peri-urban forests. It should be added, however, that in developed countries a considerable interest in forest recreation can also be observed among the rural population [4]. The significant interest in forest recreation observed over the years [3,5,6] can be explained, on the one hand, by the universality and accessibility of forests, and, on the other hand, by their beneficial effects on the health of their visitors. Forests have a positive effect on mental health [7,8,9]. Spending time in a forest has positive effects on concentration and performance [10], stress reduction [11,12], and can even aid in the treatment of depression [13,14]. Recent studies show that snowy forests can also have a positive impact on the mental state of their visitors [15,16]. Furthermore, the beneficial effects of greenery on health can be seen in the treatment of diseases such as obesity, coronary heart disease, hypertension, and hyperlipidemia [17,18,19,20,21]. Increased exposure to greenery has also been shown to reduce the risk of type 2 diabetes mellitus (T2DM) [22,23], and also to lower the risk of metabolic syndrome [24]. It has been demonstrated in laboratory conditions that camphene can be an effective drug in lowering lipid levels [25]. Tsai et al. [26] suggest that the reduced risk of developing T2DM in forest recreationists may be partly due to higher levels of terpenes in the forest air. Previously, Cho et al. [27] reported that the beneficial effects of forest bathing on human health may be attributed to compounds from the terpene group—the largest class of naturally occurring organic compounds that may have anti-inflammatory, anticancer, or neuroprotective effects. The beneficial effects of terpenes in the treatment of certain cancers were observed as early as the late 20th century [28]. Among many compounds of natural origin, monoterpenes, particularly limonene and perillyl alcohol, act both as cancer-protective agents and substances with applications in anticancer therapy. Limonene and perillyl alcohol have been shown to inhibit the development of many types of cancer, including breast, skin, liver, lung, colon, pancreatic, and prostate cancers [29,30,31,32]. Further laboratory tests confirm this efficacy [33,34], but it must be mentioned that these studies were conducted on animals or cell lines and concern selected representatives and not the entire population. It should also be borne in mind that inhalation of terpenes is generally not considered a health concern (both acute and long-term) due to their low indoor air concentrations; however, their gas and surface reactions with ozone and the hydroxyl radical produce a range of products, both gaseous, i.e. formaldehyde, and ultrafine particles, formed by condensation/nucleation processes. These reaction products may be of health concern [35].

In the literature, a large number of studies have been devoted to the beneficial effects of the forest environment on public health, a small selection of which we have quoted in the introduction. Only in a fraction of the papers did the authors attempt to answer the question of why the forest environment has such significant results in supporting the treatment of various diseases and in preventing the onset of many, with reference to terpenes present in the forest air. However, the authors of these works did not study the air’s chemical composition. Previous studies of the chemical composition of air in forests have mainly focused on the content of compounds classified as atmospheric pollutants, including especially PM10 (particulate matter with an aerodynamic diameter smaller than 10 µm) and CO_2_, along with O_3_, SO_2_, and NO_2_ [36,37]. Only a few papers have investigated terpenes in forest air considering their possible impact on public health [38,39,40,41], and, in addition, none of them have carried out a detailed quantitative and qualitative characterization of terpenes contained in the air of lowland forests of temperate-climate Europe, which may be largely responsible for the beneficial effects of the forest environment on the public health of millions of Europeans.

The aim of the pilot interdisciplinary research was to determine the chemical composition of the air inside two most common forest types of temperate-climate Europe, i.e., Nemoral Scots pine forests (the Hucisko location—Hu) and Central European submountainous beech forests (the Kłapówka location—Kl); with a particular interest in the levels of compounds from the terpene group. The results of the research can constitute a first step towards the determination of the duration of time spent in a defined type of forest (forest air inhalation time) that is needed to achieve noticeable effects of “forest treatment” of various diseases of affluence, particularly diseases of the upper respiratory tract. Research conducted by Dudek et al. [42] in these two forest types, Nemoral Scots pine forests and Central European submountainous beech forests, produced results that proved that the concentration of allergenic pollen in the forest interior is lower than in open areas and therefore there is no contraindication for allergy sufferers to enjoy outdoor recreation in forests. These results confirm earlier observations of Seo et al. [43] that forest environments can play an important role in the treatment of childhood allergic diseases. People suffering from pollen-induced allergies are often confined indoors and thus have limited opportunities for active outdoor recreation, which negatively affects their overall health. In the case of the Polish population, around 12 million people (30%) suffer from allergies (8 million have allergic rhinitis and over 4 million struggle with asthma), which can be classified among the main diseases of affluence. Forest environment can therefore constitute an opportunity for physical activity also for people with allergies, and the composition of forest air can even improve their immunity and overall health.

## 2. Materials and Methods

### 2.1. Description of Sampling Area and Location of Sampling Points

**Figure 1:**
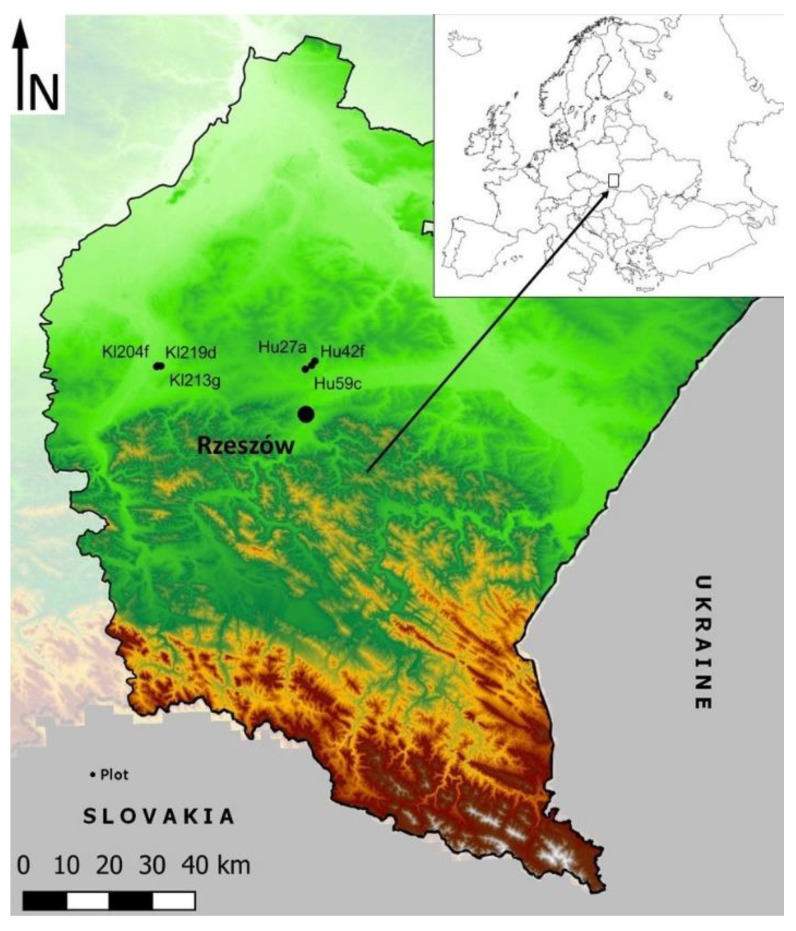
General view of a research area—southeastern Poland, Subcarpathian.

Research in forested areas was carried out from June to September 2020, in southeastern Poland in Podkarpacie (Figure 1), in the two forest types most common in the temperate-climate zone of Europe, i.e., Nemoral Scots pine forests (Hu) and Central European submountainous beech forests (Kl). The region’s forest cover is 38.3%, one of the highest in Poland, with a national average of 29.6% [44].

The climate of the region is characterized by indicators such as (1) average annual temperature: 9.7 °C; (2) total annual rain or snow precipitation: 644 mm; (3) mean growing season length: 225–230 days; (4) prevailing wind directions: S–W, W, N–W [45]. The investigated area is flat, slightly undulating, and characterized by small height differences. Elevation ranges from 205 to 235 m above mean sea level. The dominant soils are brown and podzolic soils formed on Quaternary glacial sands [46].

The following criteria were taken into consideration when selecting tree stands for the study: 3 pine stands with a pine share of at least 70% and 3 beech stands with a beech share of at least 70%; age of stands at least 70 years—older stands are characterized by a higher recreational potential [47]; distance between designated stands of at least 1 km. In this way, 6 stands were selected with the use of the Forest Data Bank (BDL; https://www.bdl.lasy.gov.pl/portal (accessed on 4 October 2020)), the locations and characteristics of which are given in Table 1.

### 2.2. Collection of Organic Compound Samples and Screening Measurements of Ozone and Atmospheric Aerosol

While collecting the air samples, the following measurements were made in the field: air temperature and air humidity were measured at a height of 1.5–1.8 m above the ground with an Elemetron PWT-401 thermohygrometer (https://elmetron.com.pl/PWT-401.html (accessed on 1 October 2022); Zabrze, Poland), 3 measurements at 1 min, at 30 min and at 60 min from the start of the stage of collecting compound samples from ambient air.

Apart from measurements made directly in the sampling areas, the following data were additionally obtained from the nearest meteorological station (Rzeszów-Jasionka, Rzeszów, Poland): atmospheric pressure, air temperature, air humidity, and wind speed (www.tutiempo.net (accessed on 1 October 2022)). Data from the station were read for the 3 times at which air sampling started on the 3 consecutive sampling plots, during each day of measurements (9 a.m., 11 a.m., 1 p.m.), and for 2 p.m. on the reference site—meteorological data from the city park of Rzeszów (see Table 2).

The representatives of VOCs, including terpenes, were collected in Tenax TA (60–80 mesh) stainless steel sorption tubes, filled with approx. 200 mg of sorbent tube (O.D. × L 1/4 in. × 3 1/2 in., preconditioned, Merck KGaA, Darmstadt, Germany). Before each sampling campaign, sorption units were cleaned and conditioned for 30 min at 300 °C under the inert gas (nitrogen) flow rate. After the conditioning, as well as the sampling process, the ends of the applied sorption tube were protected by a brass braze nut with a Teflon insert, and then the Tenax TA tube was placed in a tightly sealed polyethylene container—a more suitable solution in the case of sample transportation than glass tubes. During the sampling of analytes from the atmospheric air in the defined forest area (sampling point), the air was passed through the mentioned sampling elements filled with sorption medium employing a portable small-scale aspirator (individual aspirator type AP-8ch, S.I. “TWO-MET”, Zgierz, Poland) at a constant flow rate of approx. 150 cm^3^·min^−1^. The sampling time was set to 45 min. Each day in each investigated area, the samples of VOCs present in the atmospheric air were collected on three sorption tubes simultaneously. Each day investigations on the atmospheric air quality in the selected type of forest area were performed between 8:30 a.m. and 1:00 p.m. All samples were collected at the breathing level of an average person—sampling units were installed on a suitable tripod, i.e., 1.5–1.8 m above the ground. After sampling, the stainless steel tubes were secured for transportation to the laboratory and analyzed no longer than one week after sample collection (during this time samples were kept in a refrigerator at a temperature of 2–8 °C). Additionally, as control samples (protecting against potential damage to the sorption tubes containing analyte samples) each time at selected monitoring points, whole atmospheric air samples were collected using 3 L Tedlar bags (two Tedlar bags per sampling point). To prevent the impact of extensive sunlight and temperature exposure Tedlar bags with air samples were kept in a dark environment at room temperature.

As supplementary data and to facilitate the interpretation of the obtained research results, at the same time, the concentration of ozone in the atmospheric air in forest areas was monitored. Ozone measurements were performed with the use of a portable personal device—the ozone meter OZ-ONE (Trotec GmbH, Heinsberg, Germany). The device was installed at a height of approx. 1.5–1.8 m above the ground. The measurements were performed in three periods—1 min, 30 min, and 60 min after setting up the monitoring equipment. Additionally, the mentioned portable device has factory-built sensors for measuring air temperature and humidity. The ozone meter OZ-ONE allows the measurement of short-term exposure limits as well as time-weighted averages.

### 2.3. Laboratory Analysis—Analytical Procedure, Extraction/Liberation, and Final Determination Conditions

The separation, identification, and determination of chemical compounds collected on a sorption medium, Tenax TA, was performed according to the procedures described in detail in previous papers, with some technical modifications. In brief, to extract/liberate the analytes adsorbed on a sorption medium, Tenax TA, the thermal extraction supplied with the forced inert gas flow rate was employed—two-stage thermal desorption (TD) technique. Applied TD units were connected to two gas chromatographic (GC) systems: (i) GC equipped with a flame ionization detector (GC-FID), and (ii) GC combined with a mass spectrometer (GC-MS). From each sampling period, two Tenax TA tubes were subjected to a qualitative and quantitative analysis using a TD-GC-FID system, and one Tenax TA tube was subjected to an additional identification of the collected compounds applying the TD-GC-MS unit. General information about the working conditions of employed TD-GC-FID and TD-GC-MS systems for estimating the content level of selected representatives of organic compounds in atmospheric air on investigated forest areas is listed in Table A1.

Additionally, the abovementioned TD-GC-FID system was applied to assess the values of the TVOC parameter, which gives information about the sum of all VOCs eluting between n-hexane and n-hexadecane in the applied GC column (uncalibrated peaks are quantified in terms of toluene equivalents). Furthermore, to obtain more reliable qualitative information (organic compounds identification) for each sample the values of the linear retention index (LRI) of the determined chemical compound were calculated. The LRI value for each of the chemical compounds determined in the investigated sample was assessed based on the rule described in the literature (described by Van den Dool and Kratz [48]) that a chemical compound’s retention index is its relative time adjustment between the closest n-alkanes which leave the GC column and reaching the detector immediately before and after an investigated chemical compound [49,50]. The prepared mixture of n-alkanes on the basis of which the values of LRI were estimated contained aliphatic hydrocarbons with a carbon length from C8 (n-octane) to C17 (n-heptadecane). The mixture was analyzed under the same extraction and final determination conditions as real samples. Finally, obtained chromatograms of investigated samples and the n-alkanes mixture were analyzed and the numerical values of LRI were estimated based on Formula (1):

RI = 100n + (100 × [(TR_(x)_ − TR_(n)_)/(TR_(n+1)_ − TR_(n)_)]) (1)

As markers of atmospheric air quality in investigated forest areas, the representatives of terpene compounds were selected. The identification and quantitative analysis of collected analytes classified as terpenes were performed with the use of the external standard calibration method (ESTD). The calibration of the TD-GC-FID system and the organic compounds identification process (based on the comparison of retention times and retention indexes) was carried out using a reference standard mixture of 20 terpenes diluted in methanol at content level 2000 µg·mL^−1^ each (Cannabis Terpene Mix A certified reference material, TraceCERT^®^, Merck KGaA, Darmstadt, Germany). For each of the chemical compounds enclosed in mentioned reference standard mixture, five-point calibration curves were prepared in the concentration range from 2 to 200 ng per tube. Each calibration point was repeated three times. It was found that calculated R-square values were above 0.992, which confirms the good detector linear response. A parallel calibration procedure (with a similar concentration range of each analyte per tube) was performed to identify and determine the BTEX compounds. In this case, to carry out the calibration process of TD-GC-FID system reference standard mixture of 13 VOCs (including benzene, toluene, ethylbenzene, and p, m- xylene) diluted in methanol at content level 2000 µg·mL^−1^ each (EPA VOC Mix 2 certified reference material, TraceCERT^®^, Merck KGaA, Darmstadt, Germany) was applied. More detailed information about the calibration of TD-GC-FID was described in former papers [51,52,53]. The limit of detection (LOD) was assessed based on the average value of signal-to-noise ratio (3 × S/N) and was below 0.30 ng.

### 2.4. Statistical Analysis

The Shapiro–Wilk test was used to study the normality of the distribution. The scale in all groups is continuous. Student’s *t*-test was used to compare meteorological indices (temperature and air humidity) measured directly on the sampling plots in the forest and at the nearest meteorological station—Rzeszów-Jasionka. Pearson’s correlation coefficient was used to test the correlation between temperature and the total amount of terpenes, and between air humidity and the total amount of terpenes. The Mann–Whitney U test was used to check whether there was a statistically significant difference in the total amount of terpenes measured in Nemoral Scots pine forests and Central European submountainous beech forests and whether there was a statistically significant difference in TVOC levels recorded in Nemoral Scots pine forests and Central European submountainous beech forests.

The differences were deemed statistically significant with α = 0.05. All statistical analyses were performed with the use of Statistica v13.3 software (StatSoft Inc., Tulsa, OK, USA).

## 3. Results and Discussion

### 3.1. Meteorological Conditions over the Sampling Areas

**Figure 2:**
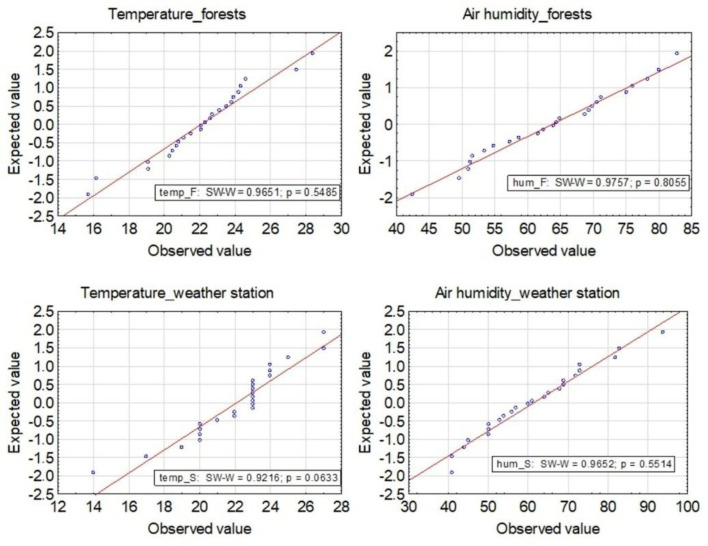
Temperature and humidity distribution of air examined by the Shapiro–Wilk test.

The results obtained for air temperature and humidity measurements in the sampling areas in the forests and the weather station are comparable (Table 2). The distribution of air temperature and humidity tested with the Shapiro–Wilk test is normal (Figure 2).

Differences between mean temperatures (*t* = 0.2608, df = 50.00, *p* = 0.7953; homogeneous variances: F = 1.1170, *p* = 0.7843) and mean humidity (*t* = 0.4764, df = 50.00, *p* = 0.6359; homogeneous variances: F = 1.3930, *p* = 0.4131) measured with the *t*-test are statistically insignificant. A negative correlation between humidity and air temperature can be observed—as temperature increases, humidity decreases (r = −0.5065, *p* = 0.012). The mean ozone concentration measured in the forest sampling plots with the exception of one measurement (15 July, Hu42f, Ozone STEL = 19 µg-m^−3^) was always below the LOQ. In contrast, the mean ozone concentration measured in the Rzeszów city park on both measurement days was 19 µg-m^−3^, with a maximum instantaneous concentration of 622 µg-m^−3^ (Table 2). A study by Peters et al. [54] showed that ozone concentrations recorded during air sampling resulted in a 10–50% decrease in terpene recovery.

### 3.2. Representatives of Volatile Organic Compounds Determined in the Investigated Forest Areas

Ambient air quality is one of the most important factors affecting human health and life quality. A special case of ambient air is forest air. People associate being in a forest with pleasant sensations, both visual and olfactory. The so-called forest scent is usually considered to be a pleasant smell, improving, among other things, our mood and general well-being, which can subsequently result in an improvement of our general health. The olfactory composition of forest air consists mainly of volatile organic compounds from the terpene and terpenoid group, emitted by the vegetation of forest complexes with varying intensity, depending, inter alia, on air temperature and humidity. An exemplary description of the odor (odor quality) associated with the presence of a particular representative of compounds from the terpene group is presented in Table A2.

The aim of the research was to study the composition of air in forest complexes with various stands. The research was carried out in six locations and air samples were collected at the level of the human respiratory zone (about 1.5 m from the surface) in order to better reflect the potential influence of forest air on the human respiratory system.

By assessing the composition of forest air in terms of the content of volatile organic compounds (based on the results of the qualitative analysis of the samples carried out with the use of TD-GC-MS), it can be concluded that the group of compounds most abundantly present in all the tested samples was aliphatic hydrocarbons, both saturated (linear and branched) and unsaturated (terpenes). The collected samples also showed the presence of carbonyl compounds (organic acids—mainly acetic acid, aldehydes, and ketones), but they constituted no more than 10% of all compounds present in the chromatograms and were not quantified in the tests performed. TVOC is a parameter that illustrates the air quality well in terms of VOC content. In the tests performed, this parameter was determined as the total content of organic compounds on the chromatograms between the C6 and C16 n-alkanes.

The group of compounds present in the forest air that received the most attention was terpenes and terpenoids. A detailed analysis of the chemical composition of the forest air samples, based on the obtained results of chromatographic analyses, allowed for the distinction, identification, and quantification of 14 compounds from the group of terpenes and terpenoids. The identification of 14 terpenes and terpenoids was carried out based on (i) the analysis of the mass spectra obtained during the TD-GC-MS analysis and their comparison with the NIST 11 Mass Spectral Database (the identification of the compound was considered satisfactory when the probability of the compound identification after comparing the live spectra with the spectra from the database was higher than 75%); (ii) the system of linear retention indices (LRI) determined using the TD-GC-FID and TD-GC-MS methods and comparing the retention times of compounds present in the samples with the retention times of the reference mixture containing the selected (identified) compounds from the group of terpenes and terpenoids. The researchers also attempted to compare the chemical composition of air in terms of the content of terpenes and terpenoids in forest complexes with different stands (Nemoral Scots pine forests (Hu) and Central European submountainous beech forests (Kl)). Information on the calculated ranges of the LRI parameter for the separated and identified terpenes and terpenoids in all studied samples of atmospheric air, along with a comparison with the literature data, is presented in Table 3.

Moreover, by examining the chemical composition of forest air in terms of the content of VOCs, the content of aromatic hydrocarbons (BTEX) was also determined, i.e., a group of compounds that constitute typical pollutants introduced into the environment as a result of human activity. The summary of the results of the qualitative and quantitative tests obtained for all locations is presented in Table 4.

**Figure 3:**
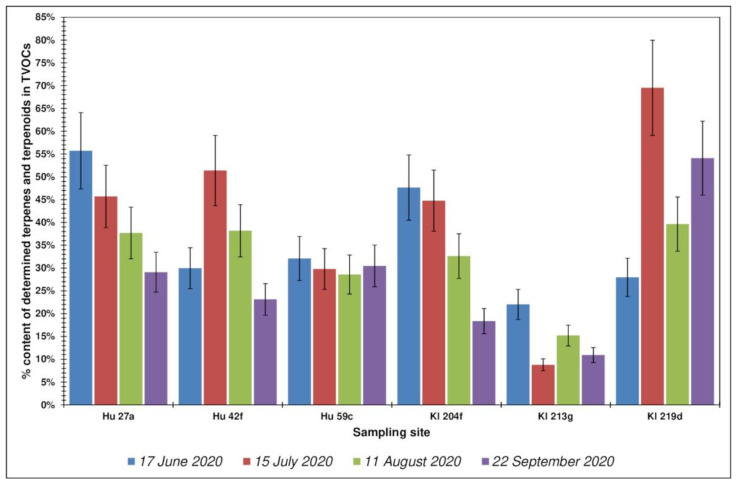
Distribution of concentrations of the sum of terpenes and terpenoids for each test site, over all measurement periods. Error bars express the standard deviation of obtained results.

**Figure 4:**
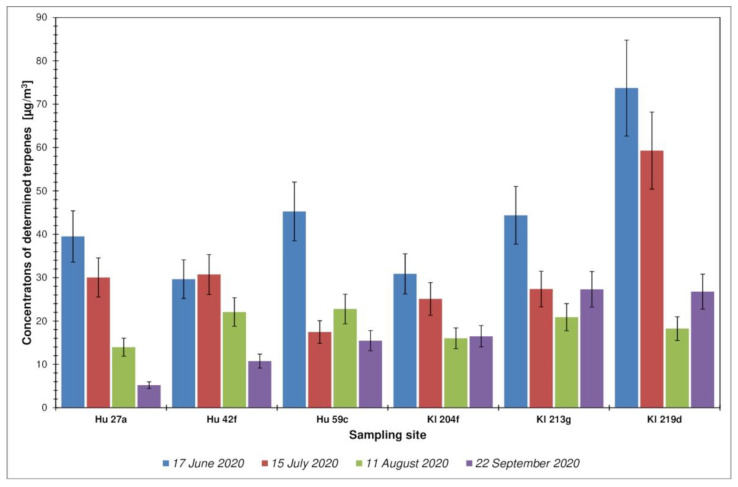
Percentage of terpenes and terpenoids in the total content of volatile organic compounds (TVOC) for each investigated site, in all measurement periods. Error bars express the standard deviation of obtained results.

Analyzing the obtained test results, it can be concluded that the contents of compounds from the group of terpenes and terpenoids in the forest air were varied and ranged between 10 and 74 µg·m^−3^ (the average value for all locations was 30.2 µg·m^−3^), which was on average approximately 33% of the total volatile organic compounds present in the tested air samples. A greater differentiation of the share of identified terpenes and terpenoids in the TVOCs was observed for the Kłapówka location (range from 9% to 70%) than for the Hucisko location (range from 9% to 59%). The distribution of total concentrations of terpenes and terpenoids for each tested location, in all measurement periods, and their percentage share in TVOCs are presented in Figure 3 and Figure 4.

**Figure 5:**
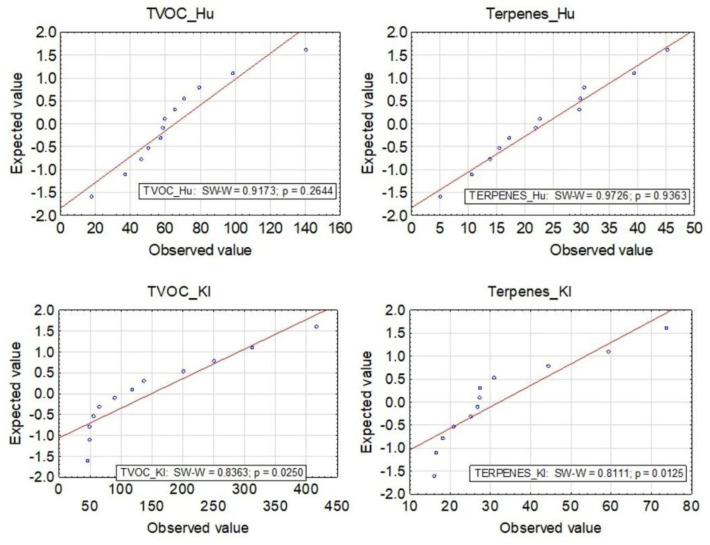
TVOC and terpene distribution investigated by Shapiro–Wilk test.

The smallest share of the identified terpenes in the total content of volatile organic compounds in all four measurement periods was observed for the forest area Kl 213g (Figure 3). In general, the highest total terpene concentration for all locations was observed in June, with the highest value of 73.7 µg·m^−3^ for Kl 219d, and the lowest value was observed in September—5.2 µg·m^−3^ in Hu 27a (Figure 4). In location Kl 219d, the highest values of total terpenes were observed during all measurement campaigns. The distribution of TVOCs and terpenes and terpenoids for the Kl location does not exhibit the characteristics of a normal distribution (Figure 5).

On the basis of the Mann–Whitney U test, it can be stated that there are no statistically significant differences in the total number of terpenes between the investigated forest types (U = 53, Z = −1.0681, *p* = 0.2855). Similarly, no statistically significant differences in the TVOCs recorded in these two types of forest can be found (U = 45, Z = −1.5300, *p* = 0.1260). Our results indicate a large variation in the content of terpenes and terpenoids in the forest air and although a beech forest might produce more terpenes than a pine forest, these differences are not statistically significant.

**Figure A1:**
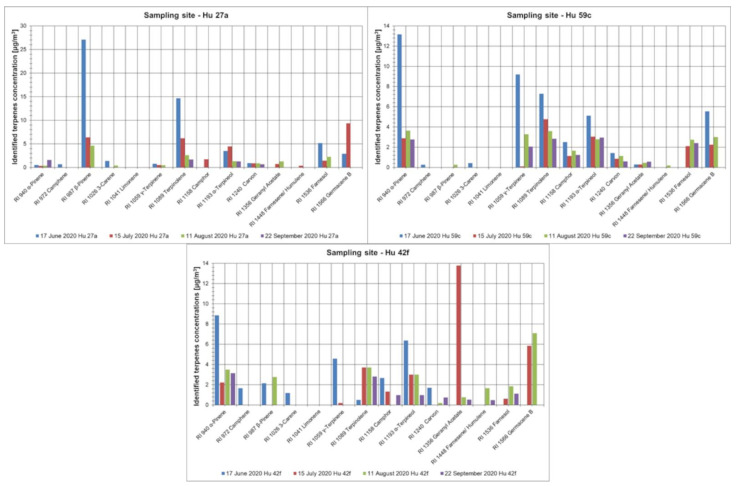
Distribution of concentrations of identified terpenes and terpenoids in atmospheric air over the Hucisko area for all measurement campaigns.

**Figure A2:**
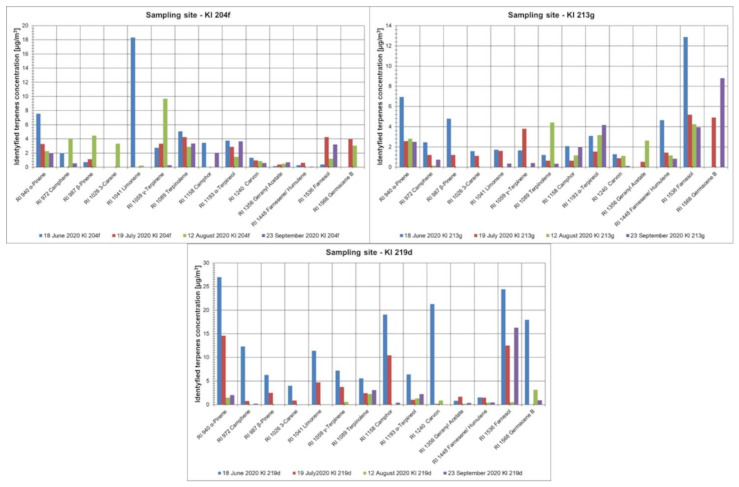
Distribution of concentrations of identified terpenes and terpenoids in atmospheric air over the Klapowka area for all measurement campaigns.

This variability is influenced by dynamically changing weather conditions: temperature, air humidity, wind speed, and direction [57,58,59]. Similarly, Meneguzzo et al. [39] indicate the variability of the concentration of biogenic volatile organic compounds (BVOCs) in time and space. However, contrary to the present study, these authors put forward a cautious conclusion that conifers are more efficient than beeches in terms of the concentration of BVOCs in the forest air. However, in the abovementioned study, the measurements were carried out along a path through various forest types, without specifying the distance between forest types, hence perhaps the BVOCs produced by one species were mixed with that produced by another. The distribution of concentrations of individual compounds from the group of terpenes and terpenoids, for each location independently, for all measurement campaigns, is presented in the figures—Figure A1 and Figure A2.

Detailed analysis of the content of compounds from the group of terpenes and terpenoids in the tested air showed that the highest concentrations of selected terpenes and terpenoids were observed in June, although the shares of individual compounds representing the group were varied. Park et al. [40], conducting year-round research in the Pinus densiflora forest in Korea, found the highest concentration of natural volatile organic compounds in summer, followed by spring, autumn, and winter. Meneguzzo et al. [39], conducting research from August to October in the Italian Apennines in coniferous (spruce, fir) and beech forests, found the highest TVOC concentration at the end of September. Interestingly, studies in the Mediterranean basin on seven tree species showed no clear seasonal trend in the emissions of terpenes and terpenoids, except for Cistus albidus, for which peak values were observed in spring and minimum values in autumn [58]. According to Meneguzzo et al. [39], detailed comparisons of the results of the analysis of the chemical composition of forest air from different forests and different latitudes do not make much sense, with which the authors agree. In the present study, there was no statistically significant relationship found between the total amount of terpenes and terpenoids and air temperature (r = 0.1048, *p* = 0.626) or between the total amount of terpenes and terpenoids and air humidity (r = 0.2947, *p* = 0.162).

The compounds that were present in all locations, during all measurement campaigns, and whose concentrations constituted a significant contribution to the total terpenes content (∑ terpenes) were: (i) α-pinene (RI 940)—% share in ∑ Terpenes for Hu was 0.6–17% (avr 9.8%), and for the Kl location—4.2–23.0% (avr 9.4%); (ii) terpinolene (RI 1089)—for Hu it was 1.2–33.2% (avr 12.8%), and for the Kl location—2.0–13.9% (avr 7.9%); (iii) α-terpineol (RI 1193), for Hu it was 5.4–15.3% (avr 8.8%), and for the Kl location—1.7–14.3% (avr 7.6%).

Additionally, for the Kl location, farnesol (RI 1536) was an important compound from the group of terpenes and terpenoids; its share in the total content of terpenes and terpenoids was in the range of 0.7–48.6%, and the mean value was 12.8% (see Figure A2).

A significant difference in the profile of terpene compounds present in the forest air of the studied locations is the absence of limonene in the Hu 27a, Hu 42f, and Hu 59c locations during all measurement campaigns (see Figure A1).

**Figure 6:**
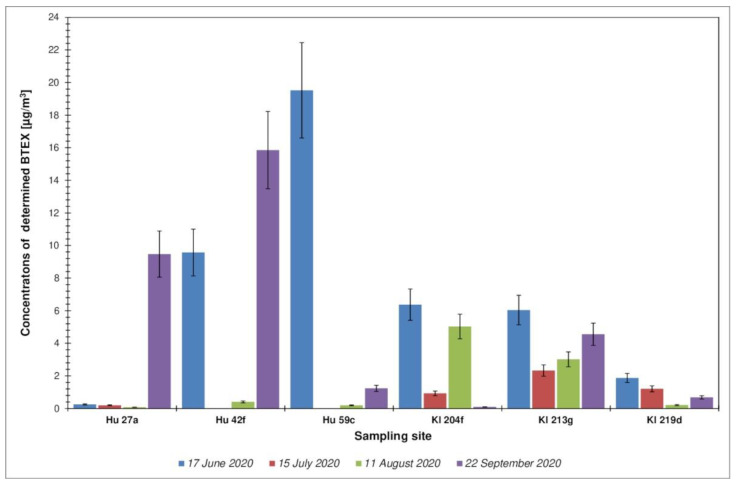
Total content of BTEX compounds determined for all locations and all measurement campaigns. Error bars express the standard deviation of obtained results.

Another group of chemical compounds that was monitored was the BTEX group (benzene, toluene, ethylbenzene, p-, m-xylene). When assessing the concentrations of BTEX compounds in the forest air, it can be concluded that they were also varied. It should be emphasized, however, that the concentrations of individual representatives of the BTEX group in no case exceeded the permissible values for atmospheric air (it is particularly important that the concentration of benzene was below the permissible value of 5 µg·m^−3^; Directive 2008/50/EC of the European Parliament and of the Council of 21 May 2008 on air quality and cleaner air for Europe). The total BTEX content for all locations and all measurement campaigns is shown in Figure 6.

The concentration values of individual compounds from the BTEX group are presented in the aforementioned Table 4. It is worth noting that the compound with the largest share in the total BTEX content was toluene, a compound with a lower toxicity than benzene. This state is not surprising because in the vast majority of cases toluene is recorded in the atmospheric air in open spaces at a much higher level than benzene [60,61,62]. In the case of the area where the research discussed in the present paper was carried out, the level of benzene content in the forest air ranged from <0.014 to 2.712 µg·m^−3^ (Table 4). For comparison, the concentration of benzene in the atmospheric air in Berlin was 6.9 µg·m^−3^ [63], and in 13 sites in 8 US states it was 0.8–3.6 µg·m^−3^ [64]. In the case of atmospheric air in the area of a city considered a recreational and health resort—Sopot, Poland—the concentration of benzene in 2013 and 2014 was 0.53 ± 0.43 µg·m^−3^ and 1.07 ± 0.97 µg·m^−3^, respectively [65]. The presence of BTEX chemicals in the atmospheric air is mainly a consequence of various types of human activity, especially the movement of mechanical vehicles powered by internal combustion engines and activities of different types of industrial centers. The presence of these compounds in the monitored forest areas (far away from the main communication routes or industrial centers) may be caused by the movement of air masses and their movement over long distances to the monitored green areas. Low levels of xylene and ethylbenzene in the atmospheric air in forest areas could constitute confirmation of this hypothesis, since they indicate the presence of the so-called old air masses—compounds determined in forest air may come from distant emission sources, e.g., urban agglomerations, industrial plants, and main communication routes [65,66].

### 3.3. Aerosol Concentration in Forest Air

**Figure 7:**
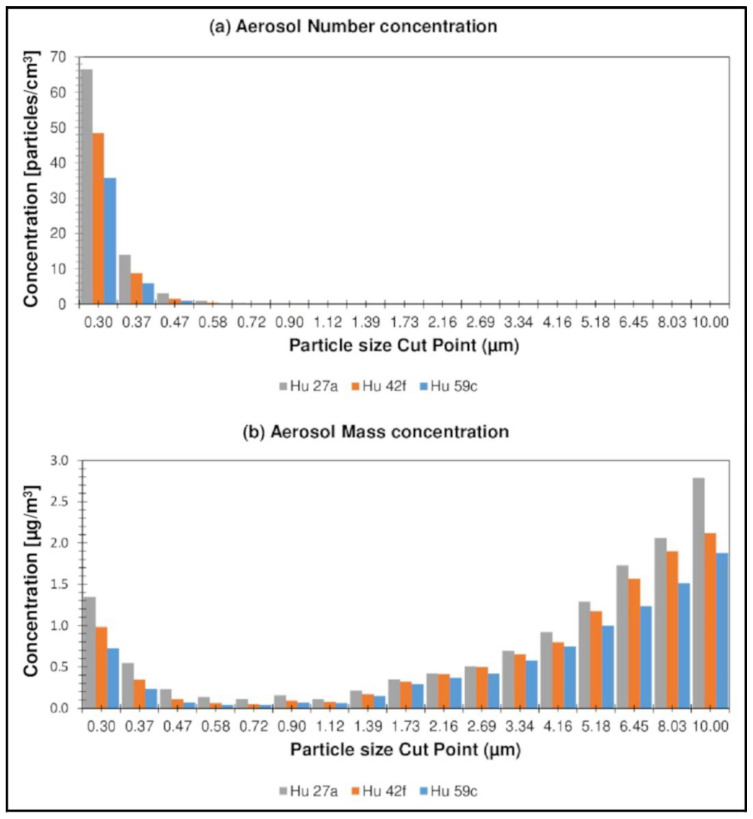
Taking into account the distribution of aerosol particle diameters, for Nemoral Scots pine forests.

**Figure 8:**
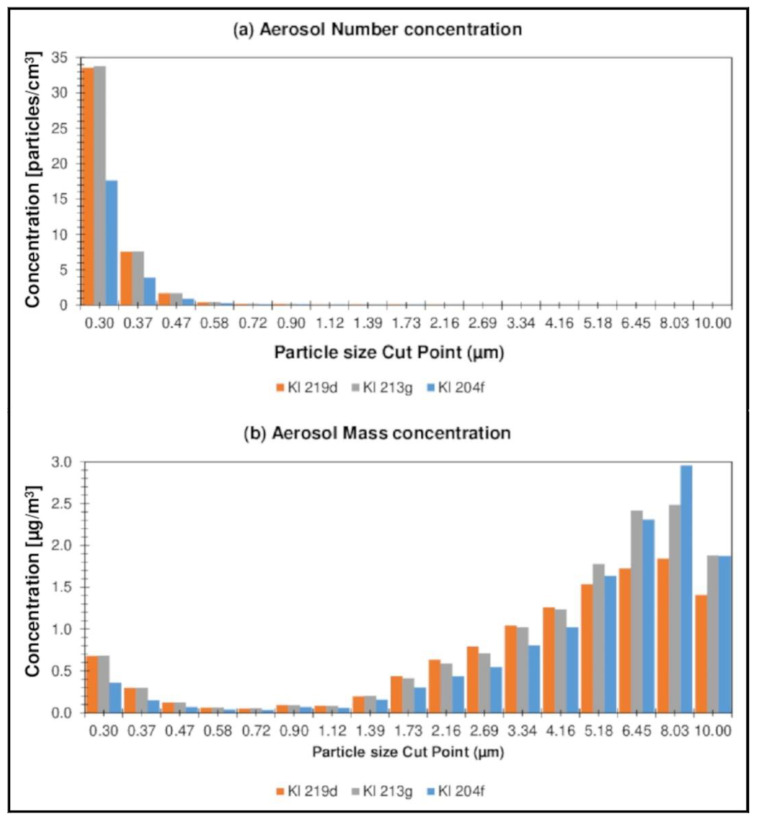
Taking into account the distribution of aerosol particle diameters, for Central European submountainous beech forests.

In order to make a more complete characterization of forest air, the aerosol concentration was also assessed during the August measurement campaign. The aerosol content (both the number and the mass concentration of the aerosol) was determined in the particle diameter range from 0.3 to 10 µm. The obtained results, representing the number and the mass concentration of the aerosol, taking into account the distribution of the aerosol particle diameters, are presented separately for Nemoral Scots pine forests (Figure 7a,b) and Central European submountainous beech forests (Figure 8a,b).

Higher values of concentrations were observed for the Hu forest complexes, the highest value being 85.7 particles/cm^3^ (Hu 27a). The highest mass concentration of atmospheric aerosol was observed for the Kl 213g location and was 14.3 µg·m^−3^. At Hu locations, the share of particles with smaller diameters (0.3–0.5 µm) was more significant in the total distribution of aerosol particles than for Kl forest complexes, which resulted in a higher value of the number concentration and a lower value of the mass concentration; nevertheless, the measured values of the number and the mass concentration aerosol levels in forest air in all locations are lower than those described in the literature mainly for the urban environment [67,68] and are considered to be “neutral level” (considered the level acceptable for clean air).

The concentration of aerosol in forest air varies and depends, among other things, on the concentration of terpenes and other compounds from the group of reactive organic compounds, tropospheric ozone, and humidity. Moreover, it should be emphasized that the conducted measurements of atmospheric aerosol concentration are illustrative measurements intended to be used for the general characterization of forest air, and not for comparison with the applicable regulations. Terpenes in the atmosphere are reactive compounds that take part in the formation of atmospheric aerosols in the presence of atmospheric oxidants such as hydroxyl radicals, tropospheric ozone, or nitrogen oxides; however, our research on the level of atmospheric aerosol content in forest air is meant to be used to describe the quality of forest air more fully in terms of influencing the human respiratory system.

## 4. Conclusions

Our results indicate a large variation in the content of terpenes and terpenoids in the forest air (ranging between 10 and 74 µg·m^−3^) and although a beech forest might produce more terpenes than a pine forest, these differences are not statistically significant. A significant difference in the profile of terpene compounds present in the forest air of the studied locations is the absence of limonene in the pine forest during all measurement campaigns. The highest total terpene concentration for all locations was observed in June (73.7 µg·m^−3^ for Kl 219d), and the lowest value was observed in September (5.2 µg·m^−3^ in Hu 27a).

The compounds that were present in all locations, during all measurement campaigns, and whose concentrations constituted a significant contribution to the total terpenes content were α-pinene (RI 940), terpinolene (RI 1089), and α-terpineol (RI 1193); additionally, for the Kl location, farnesol (RI 1536).

The air of the studied forest types contains less benzene (<0.014 to 2.712 µg·m^−3^) and aerosol particles (to 14 µg·m^−3^) than urban air.

Considering all aspects related to the conducted research and the obtained research results, it can be concluded that the presented characteristics of forest air, taking into account compounds of both natural (quantitative and qualitative terpenes) and anthropogenic origin (found in forest air), will prompt a wide interdisciplinary discussion on the possibility of incorporating “forest treatment” into general public health management programs. The forest atmosphere has a positive effect on building immunity [69,70], which is particularly important in terms of public health (mental and physical) and can be an effective tool supporting healthcare services in the fight not only against diseases of affluence but also with periodically emerging pandemics, e.g., COVID-19. Research [71] proves that monoterpenes, including e.g., α-pinene, easily transfer from the forest air through the respiratory tract into the body and are accumulated in the serum. Thus, we believe that with the appropriate medical knowledge, and with the results published by us, we can effectively implement forest therapy in Nemoral Scots pine forests and Central European submountainous beech forests as a method of supporting the pharmacological treatment of many diseases, especially of the respiratory system.

## Figures and Tables

**Table 1:** Characteristics of stands parameters based on the forest management plan [46] and location of research plots.

Forest District Units	Coordinates (°)	Area (ha)	SC (%)	Admixture Species	Age (Years)	UU (%)	H (m)
Hu27a	N50.14072 E22.03605	7.51	100P	Q, Pi, A, F, C	89	70	25
Hu42f	N50.13173 E22.02485	8.53	100P	B	77	20	21
Hu59c	N50.12437 E22.00373	18.17	100P	Q, B	78	30	22
Kl204f	N50.14303 E21.52324	7.72	90F; 10Ac	Pi, C	79	10	27
Kl213g	N50.14227 E21.53394	2.50	70F; 30Q	-	76	10	29
Kl219d	N50.14136 E21.52017	4.29	80F; 20Q	Ac, C	86	30	29

Hu: forest district Hucisko; Kl: forest district Kłapówka; SC: tree species composition; P: Pinus sylvestris; Q: Quercus robur; F: Fagus sylvatica; C: Carpinus betulus; A: Abies alba; B: Betula pendula; Pi: Picea abies; Ac: Acer pseudoplatanus; UU: undergrowth and underbrush; H: height of tree stands according to the dominant species.

**Table 2:** Results of meteorological and tropospheric ozone measurements.

Date	Forest District Units	Land Field Measurements				Data from Meteo Station Rzeszów-Jasionka			
		T (°C) (avg.)	H (%) (avg.)	Ozone (avg.) * (µg·m^−3^)	Ozone (Min–Max) (µg·m^−3^)	AP (hPa)	T (°C) (avg.)	H (%) (avg.)	W (km/h) (avg.)
17.06	Hu27a	21.1	75.1	<LOQ	<LOQ	1009	20	83	7
	Hu42f	24.3	64.5	<LOQ	<LOQ	1008	22	73	7
	Hu59c	23.8	64.9	<LOQ	19–335	1008	23	69	15
18.06	Kl204f	22.3	76.1	<LOQ	19–39	1008	19	94	6
	Kl213g	20.7	82.9	<LOQ	<LOQ	1008	23	69	22
	Kl219d	23.9	71.3	<LOQ	<LOQ	1007	24	65	20
15.07	Hu27a	20.5	51.6	<LOQ	19–270	1015	20	60	7
	Hu42f	22.1	49.6	19	19–510	1014	22	50	13
	Hu59c	24.6	42.4	<LOQ	19–195	1014	24	41	0
	Urban area	26.0	36.5	19	19–622	1014	23	41	6
16.07	Kl219d	20.3	69.4	<LOQ	<LOQ	1012	21	64	13
	Kl213g	22.6	53.5	<LOQ	<LOQ	1012	23	53	20
	Kl204f	22.7	57.3	<LOQ	19–19	1012	23	61	22
	Urban area	24.3	52.9	19	19–409	1012	22	60	26
11.08	Hu27a	24.2	69.9	<LOQ	<LOQ	1019	23	73	2
	Hu42f	27.5	58.7	<LOQ	<LOQ	1019	27	54	6
	Hu59c	28.4	51.0	<LOQ	<LOQ	1018	27	45	9
12.08	Kl219d	19.1	78.3	<LOQ	<LOQ	1020	20	68	4
	Kl213g	21.5	68.7	<LOQ	<LOQ	1020	23	57	13
	Kl204f	23.1	61.7	<LOQ	19–158	1020	24	41	13
22.09	Hu27a	15.7	70.6	<LOQ	<LOQ	1019	14	82	2
	Hu42f	19.1	64.0	<LOQ	<LOQ	1018	20	56	13
	Hu59c	22.1	54.9	<LOQ	<LOQ	1018	23	50	13
23.09	Kl219d	16.2	80.0	<LOQ	<LOQ	1012	17	72	4
	Kl213g	20.8	62.4	<LOQ	<LOQ	1012	23	50	15
	Kl204f	23.5	51.3	<LOQ	<LOQ	1011	25	44	15

T—air temperature; H—air humidity; AP—atmospheric pressure; W—wind speed. * indicates the average value of the ozone concentration measured over a period of 15 min.

**Table 3:** Calculated retention indices of terpenes determined in atmospheric air on the investigated forest areas.

Chemical Compound	CAS No.	Formula	Range of LRI on DB-1 for Investigated Samples Based on GC-FID Analysis	Range of LRI on HP-1 MS for Investigated Samples Based on GC-MS Analysis	Range of LRI Based on Literature Data on Similar GC Column ^(a)^
α-Pinene	80-56-8	C_10_H_16_	949–956	940–946	927–932
Camphene	79-92-5	C_10_H_16_	969–975	955–960	940–946
β-Pinene	127-91-3	C_10_H_16_	996–999	982–987	969–974
3-Carene	13466-78-9	C_10_H_16_	1025–1031	1014–1020	1002–1004
Limonene	5989-27-5	C_10_H_16_	1039–1045	1031–1036	1024–1026
γ-Terpinene	99-85-4	C_10_H_16_	1064–1070	1057–1062	1054–1056
Terpinolene	586-62-9	C_10_H_16_	1101–1106	1082–1086	1085–1090
Camphor	464-49-3	C_10_H_16_O	1160–1166	1158–1063	1141–1148
α-Terpineol	98-55-5	C_10_H_18_O	1182–1188	1170–1176	1186–1194
Carvone	99-49-0	C_10_H_14_O	1248–1254	1231–1236	1239–1248
Geranyl Acetate	105-87-3	C_12_H_20_O_2_	1361–1367	1363–1369	1379–1385
Farnesene/Humulene	6753-98-6	C_15_H_24_	1474–1480	1443–1450	1440–1452
Nerolidol/Farnesol	7212-44-4	C_15_H_26_O	1531–1537	1527–1533	1531–1542
Germacene B	15423-57-1	C_15_H_24_	1561–1568	1558–1563	1559–1565

^(a)^ RI values of essential oil components for GC HP-5 MS column based on literature data from Bottoni et al. [55] and Khan et al. [56].

**Table 4:** Concentrations of selected organic compounds determined in samples collected during all sampling campaigns in the forest atmosphere: (i) TVOC—sum of concentrations of all VOC present in the sample; (ii) concentration of BTEX compounds and the sum of BTEX compounds; (iii) the sum of identified terpenes/terpenoids.

	Sampling Date	TVOC (µg/m^3^)	∑TERPENES (µg/m^3^)	Benzene (µg/m^3^)	Toluene (µg/m^3^)	Ethylbenzene (µg/m^3^)	p, m-Xylene (µg/m^3^)	ΣBTEX (µg/m^3^)
Hu 27a	17 June 2020	70.9	39.5	0.098	0.152	<LOQ	<LOQ	0.25
Hu 27a	15 July 2020	65.7	30.0	<LOQ	0.151	<LOQ	0.045	0.20
Hu 27a	11 August 2020	37.0	13.9	<LOQ	0.079	<LOQ	<LOQ	0.08
Hu 27a	22 September 2020	17.9	5.2	1.011	7.469	<LOQ	0.993	9.5
Hu 42f	17 June 2020	98.9	29.7	0.469	9.102	<LOQ	<LOQ	9.6
Hu 42f	15 July 2020	59.8	30.7	<LOQ	<LOQ	<LOQ	<LOQ	<LOQ
Hu 42f	11 August 2020	57.8	22.1	0.346	0.060	<LOQ	<LOQ	0.41
Hu 42f	22 September 2020	46.5	10.8	0.571	11.961	2.967	0.349	16
Hu 59c	17 June 2020	141.0	45.3	0.934	18.586	<LOQ	<LOQ	19
Hu 59c	15 July 2020	58.6	17.4	<LOQ	<LOQ	<LOQ	<LOQ	<LOQ
Hu 59c	11 August 2020	79.7	22.8	<LOQ	<LOQ	<LOQ	0.192	0.19
Hu 59c	22 September 2020	50.8	15.5	0.358	0.037	<LOQ	0.843	1.2
Kl 204f	18 June 2020	64.8	30.9	2.094	1.983	<LOQ	2.295	6.4
Kl 204f	19 July 2020	56.0	25.1	0.623	<LOQ	<LOQ	0.306	0.93
Kl 204f	12 August 2020	49.1	16.0	<LOQ	2.297	1.343	1.390	5.0
Kl 204f	23 September 2020	89.8	16.5	<LOQ	<LOQ	<LOQ	<LOQ	<LOQ
Kl 213g	18 June 2020	201.5	44.4	2.141	0.937	<LOQ	2.961	6.0
Kl 213g	19 July 2020	311.8	27.4	<LOQ	0.910	0.369	1.053	2.3
Kl 213g	12 August 2020	137.2	20.9	0.965	0.184	<LOQ	1.870	3.0
Kl 213g	23 September 2020	250.1	27.3	2.712	0.011	<LOQ	1.834	4.56
Kl 219d	18 June 2020	263.6	73.7	0.237	0.646	0.641	0.344	1.87
Kl 219d	19 July 2020	117.9	59.3	0.563	0.084	0.040	0.519	1.21
Kl 219d	12 August 2020	46.0	18.2	<LOQ	0.209	<LOQ	<LOQ	0.21
Kl 219d	23 September 2020	49.5	26.8	0.517	0.074	<LOQ	0.094	0.68

LOQ—0.014 µg·m^−3^.

## Data Availability

Not applicable.

## Appendix A

**Table A1:** Thermal desorption (TD) GC-FID and GC-MS system parameters used to assess the content level of selected representatives of organic compounds in atmospheric air in the investigated forest areas.

Equipment Type Employed to Perform Analytes Thermal Extraction		
System Acronym	TD-GC-FID	TD-GC-MS
Type of thermal desorption unit	Markes’ Series 2 Thermal Desorption System; UNITY/TD-100	Markes Unity v.2
Analyte sampling device	Sorption tubes filled with Tenax TA (O.D. × L ¼ in. × 3 ½ in.)	
**1st stage of thermal desorption process—working conditions**		
Heating temperature	290 °C	
Inert gas (He) flow rate during the desorption stage	50 mL·min^−1^	
Heating time	15 min	
Microtrap composition and temperature	Microtrap dedicated to measuring monoterpene compounds filled with Tenax TA and Carbotrap; 0 °C	
**2nd stage of thermal desorption process—working conditions**		
Microtrap temperature (ballistic heating mode)	300 °C	
Microtrap heating time	5 min	
Flow rate of the inert gas (He) through the microtrap to the chromatographic column	2.0 mL·min^−1^	1.0 mL·min^−1^
**Final determination system working parameters**		
Type of applied gas chromatography	Agilent 7820A GC	Agilent Technologies 6890
Detector	Flame ionization detector, detector temp. 280 °C	Mass spectrometer (5873 Network Mass Selective Detector, Agilent Technologies); transmission line temp. of GC-MS: 285 °C; ion source temp.: 230 °C; quadrupole mass analyzer temp.: 150 °C
TD-GC transfer line temperature	160 °C	
Capillary column type	DB-1 (J&W, USA); 30 m × 320 µm × 5 μm	HP-1 ms (J&W, USA); 30 m × 0.25 µm × 1 μm
Helium gas (flow rate)	2.0 mL·min^−1^	1.0 mL·min^−1^
Oven temperature program	50 °C for 1 min; 7 °C·min^−1^ up to 260 °C and held for 6 min	
Data processing system	OpenLAB CDS ChemStation Workstation VL	Chemstation

**Table A2:** Some basic information on scent and chemical structure of terpene/terpenoid compounds identified/present in the studied forest atmosphere.

Retention Index/Chemical Name	Scent/Odor Quality	Chemical Structure	Formula
RI 950 α-Pinene	fir needle-like, resin-like	Bicyclic	C_10_H_16_
RI 972 Camphene	terpene-like, woody type	Bicyclic	C_10_H_16_
RI 987 β-Pinene	woody-green pine-like	Bicyclic	C_10_H_16_
RI 1026 3-Carene	terpene-like	Bicyclic	C_10_H_16_
RI 1041 Limonene	citrus-like	Cyclic,	C_10_H_16_
RI 1059 γ-Terpinene	petrol-like	Cyclic,	C_10_H_16_
RI 1089 Terpinolene	pine-like	Cyclic	C_10_H_16_
RI 1160 Camphor	camphor-like	Ketone, cyclic	C_10_H_16_
RI 1193 α-Terpineol	flowery, citrus-like, pleasant odor similar to lilac	Alcohol, cyclic	C_10_H_18_O
RI 1240 Carvone	mint-like, caraway-like	Ketone	C_10_H_14_O
RI 1356 Geranyl Acetate	pleasant floral or fruity rose aroma	Ester, linear	C_12_H_20_O_2_
RI 1448 Farnesene/Humulene	sweet, flowery/balsamic vinegar-like/woody type	Linear/cyclic, sesquiterpene	C_15_H_24_
RI 1536 Nerolidol/Farnesol	citrus-like, flowery	Alcohol, linear, sesquiterpene	C_15_H_26_O
RI 1566 Germacene B	woody type odor	Cyclic, sesquiterpene	C_15_H_24_
